# The Role of Autobiographical Memory in the Development of a Robot Self

**DOI:** 10.3389/fnbot.2017.00027

**Published:** 2017-06-20

**Authors:** Gregoire Pointeau, Peter Ford Dominey

**Affiliations:** ^1^Institut National de la Santé et de la Recherche Médicale, Stem Cell and Brain Research Institute U1208, Univ Lyon, Université Claude Bernard Lyon 1Lyon, France; ^2^Robot Cognition Laboratory, Centre National de la Recherche ScientifiqueLyon, France

**Keywords:** human-robot interaction, autobiographical memory, self

## Abstract

This article briefly reviews research in cognitive development concerning the nature of the human self. It then reviews research in developmental robotics that has attempted to retrace parts of the developmental trajectory of the self. This should be of interest to developmental psychologists, and researchers in developmental robotics. As a point of departure, one of the most characteristic aspects of human social interaction is cooperation—the process of entering into a joint enterprise to achieve a common goal. Fundamental to this ability to cooperate is the underlying ability to enter into, and engage in, a self-other relation. This suggests that if we intend for robots to cooperate with humans, then to some extent robots must engage in these self-other relations, and hence they must have some aspect of a self. Decades of research in human cognitive development indicate that the self is not fully present from the outset, but rather that it is developed in a usage-based fashion, that is, through engaging with the world, including the physical world and the social world of animate intentional agents. In an effort to characterize the self, Ulric Neisser noted that self is not unitary, and he thus proposed five types of self-knowledge that correspond to five distinct components of self: ecological, interpersonal, conceptual, temporally extended, and private. He emphasized the ecological nature of each of these levels, how they are developed through the engagement of the developing child with the physical and interpersonal worlds. Crucially, development of the self has been shown to rely on the child's autobiographical memory. From the developmental robotics perspective, this suggests that in principal it would be possible to develop certain aspects of self in a robot cognitive system where the robot is engaged in the physical and social world, equipped with an autobiographical memory system. We review a series of developmental robotics studies that make progress in this enterprise. We conclude with a summary of the properties that are required for the development of these different levels of self, and we identify topics for future research.

## Introduction

While the notion of self is so ubiquitous in our experience, the development and origin of self is a complex process that is not yet fully understood (Damasio, 2012; Gallagher, 2013). Self is at the core of the self-other relation that defines human social interaction. In this context of social interaction, Tomasello and his colleagues have studied the development of human cooperation (Tomasello, 2000; Tomasello et al., 2005). They discovered that there is a fundamental and intrinsic motivation for children to share mental states. These mental states can include sharing objectives and sharing the plans to reach these objectives. These shared intentions and plans underlie the human capability to cooperate, and require the notion of self and other. On the basis of this self-other relation, there must be a self. This poses the question defining the self. In this context Ulric Neisser proposed a theoretical context to understand the notion of “self” that provided a highly cited basis for structuring thought and research about the self (Neisser, 1988). He argued that the self is not a unitary process, and he proposed five distinct levels of self and self-knowledge:

1. The Ecological Self: is the individual situated in and acting upon the immediate physical environment.
2. The Interpersonal Self: is the individual engaged in social interaction with another person.
3. The Conceptual Self: or self-concept, is a person's mental representation of his/her own (more or less permanent) characteristics.
4. The Temporally extended self is the individual's own life-story as he/she knows it, remembers it, tells it, projects it into the future.
5. The Private Self defined such as it appears when the child comes to understand and value the privacy of conscious experience; when it becomes important that no one else has access to his/her thoughts, dreams, and interpretations of experience. This level of self will not be addressed in the current review.

In his 1976 book “Cognition and Reality” (Neisser, 1976), Neisser argued that the self should be considered in its ecological context. Indeed, Neisser rejected the experimental method of his peers which he considered too focused on laboratory experiments. He thus started to develop the idea of the importance of the ecological aspect of cognition. Ecological should here be interpreted as “coherent” in a “natural way” (which implies the environment, the body state, and the cultural state). This ecological approach includes a focus on the importance of experience in the construction of self.

Extensive research in cognitive development demonstrates the important link between experience encoded in autobiographical memory and self (Nelson, 2003b; Nelson and Fivush, 2004). Autobiographical memory provides a structured repository for experience that is the basis of higher cognitive function in man, including aspects of language and the emergence of self (Nelson and Fivush, 2004). Indeed Neisser himself notes that “The notion that the sense of self depends on autobiographical memory is hardly new” (p. 396) (Neisser, 1988). From this perspective where we consider Neisser's progressive levels of self, and an autobiographical memory capability that can store and organize experience, a new insight emerges, whereby autobiographical memory allows the structured accumulation of experience that contributes to this development of self. In this context, the objective of this review is to provide an overview of how developmental robotics studies that rely on autobiographical memory can accomplish to certain degrees the first four levels of self-identified by Neisser.

From this perspective, research in developmental robotics and cognitive system can contribute to the pursuit of understanding human cognitive development, and through a complimentary interaction this should contribute to more functional robot systems. The developmental robotics perspective holds that certain types of behaviors are better learned than pre-programmed (Asada et al., 2009). This is particularly true in the case of social behavior and social conventions. Adapting to the context of family, school, culture, etc. requires the individual to accommodate the constraints inherent in these different domains.

## Autobiographical memory for development of a robot self

As reviewed above, autobiographical memory plays an important role in the child's development of self in these social contexts (Neisser, 1988; Nelson, 2003b; Nelson and Fivush, 2004). This emphasizes the importance of the auto-biographical memory (ABM) as the structured repertory from which self can be constructed. However, if the ABM is just a record of experienced events, it will be of little use in cognitive development. The stored knowledge must be made meaningful. From the robotics perspective, the challenge would be to implement an ABM system that encodes experience but also makes it meaningful, so that it can contribute to the construction of the self. We thus developed an ABM and corresponding reasoning capabilities for the robot to manipulate its autobiographical memory, create new knowledge, and re-organize the memory (Pointeau et al., 2014). Here we briefly review the ABM and associated reasoning capabilities, and then review studies that indicate how this ABM capability allowed the emergence of the different levels of self-defined by Neisser.

This work is situated in the developmental robotics context where the iCubLyon01 robot has undergone extensive interaction with humans using complex sensorimotor and cognitive systems over extended time (several years). This extended interaction with people provides contents for an extended history of experience that was encoded in an autobiographical memory (ABM) (Pointeau et al., 2013, 2014) that encoded (1) the history of interactions, thus suitable for learning actions and shared plans, and (2) extensive information from which regularities (e.g., names of locations, objects, action pre- and post-conditions) were extracted through a process of consolidation. This provided a framework of cognitive functions from which we could begin to take the next steps, toward development of self, based on autobiographical memory.

### Psychologically motivated autobiographical memory

**Figure 1:**
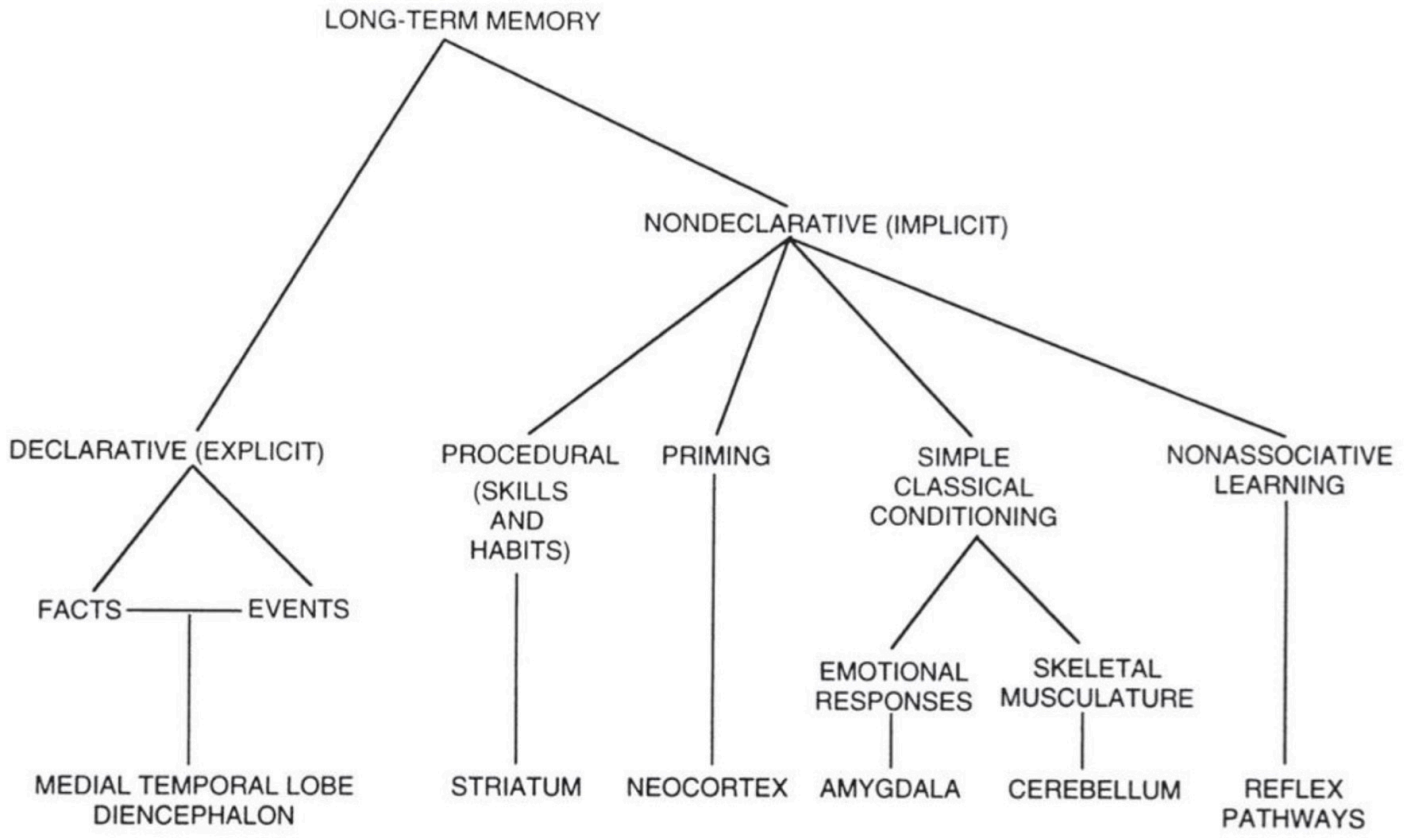
Taxonomy of the division of mammalian long term memory systems. From Squire (2004).

In human developmental studies, significant attention has been allocated to the mechanisms that underlie the ability to acquire and build knowledge and encode the individual's accumulated experience, and to use this accumulated experience to adapt to novel situation (Wells, 1981; Nelson, 2009; Kolb, 2014). We have thus recognized the adaptive importance of long-term memory in the development of self. Long-term memory can be considered in terms of declarative and non-declarative memory (Squire, 2004). Simplifying, the declarative memory is related to higher cognition, where the non-declarative memory is dedicated to actions and behaviors (see Figure 1).

Squire goes further and employs the term “representational” for the declarative memory and justifies this by the fact that declarative memory is a model of the world and thus can be right or wrong as any model. The non-declarative on the other hand cannot be wrong or false, it “is” and the recall of information is done “by rote.” Cohen and Squire (1980) also define the declarative memory as “a flexible memory for past events and facts” and we focused on this definition in the development of our model of autobiographical memory. This declarative memory of the robot was further divided in two components: episodic memory and semantic memory. The episodic memory stores all the events witnessed by the robot within a context. Each time before and after the robot performed an activity (that includes but is not restricted to: physical action—speech production or comprehension—human action recognition—reasoning) the robot took a “snapshot” of the perceived state of the world, and stored this chronologically in the episodic memory. The robot could thus retrieve a specific memory and the state of the world at a given time.

**Figure 2:**
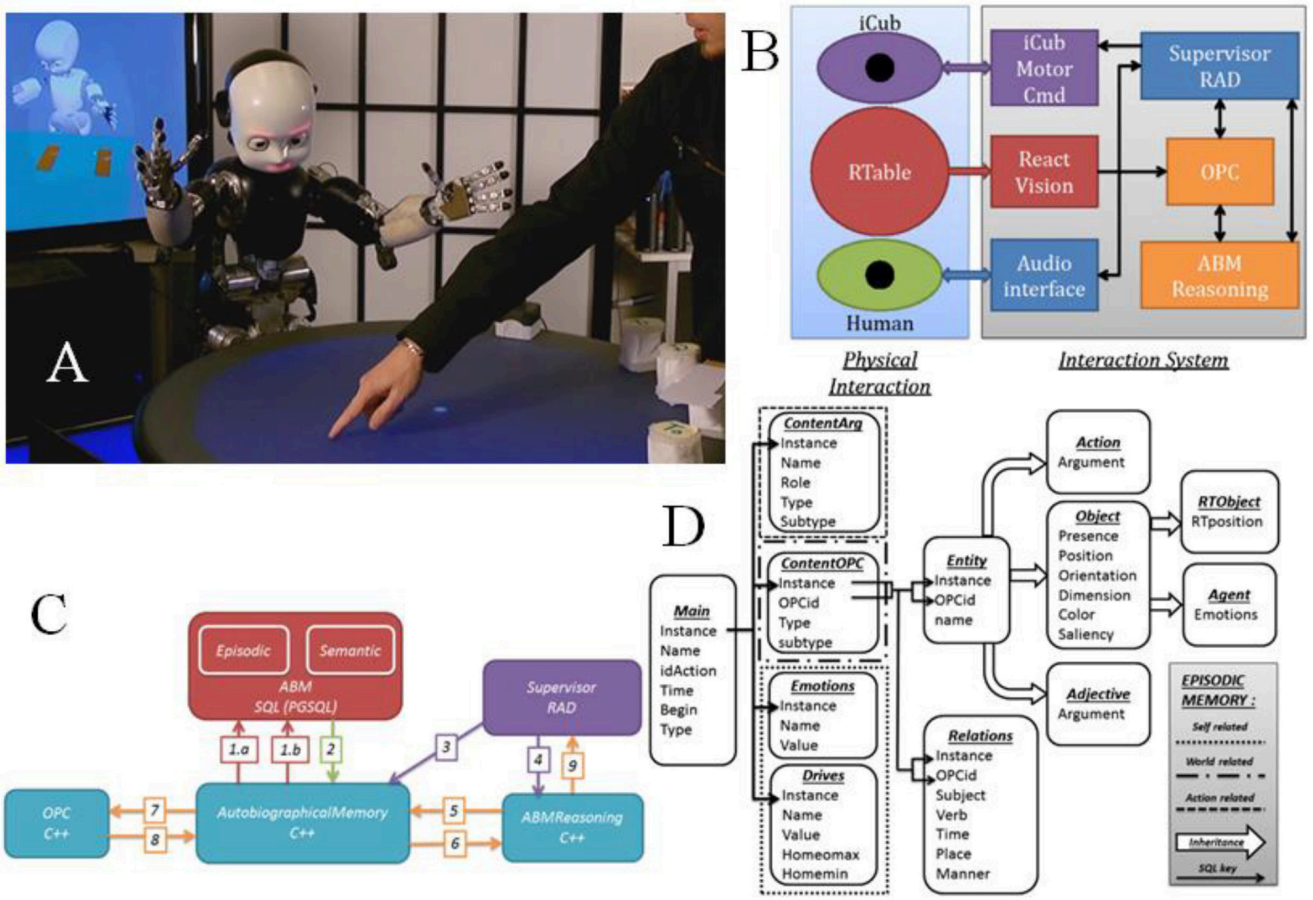
**(A)** iCub robot interacting with the human. Mental model displayed on screen in upper left. **(B)** System architecture overview. Human and iCub interact face-to-face across the ReacTable, which communicates object locations via ReactVision to the object property collector (OPC). The Supervisor coordinates spoken language and physical interaction with the iCub via spoken language technology in the Audio interface. The autobiographical memory system encodes world states and their transitions due to human and robot action as encoded in the OPC. **(C)** Overview of the memory functioning including the ABM SQL Database, the Supervisor, the Reasoning module, and the OPC. This provides a partial zoom in on **(B)**. **(D)** Architecture of the episodic memory storage in PostgreSQL. The main data type is specified as ContentArg, ContentOPC. Each interaction has the content of the OPC at a given time (state of the world), but also, information concerning the context of the action (who, what, when…). The content of a memory can be divided in three sections: self-related, world-related, and action-related. From Pointeau et al. (2014).

Figure 2 provides details of the ABM architecture and its functioning. The ABM is implemented as an SQL database and a set of C++ functions that access and process the ABM contexts. In Figure 2A the human and robot interact in a physical space, with actions involving objects, and they interact socially via spoken language. Figure 2B illustrates the system architecture, where information about actions performed on objects is perceived by ReactVision™ and updates the world model in the Object Property Collector (OPC), which feeds into the ABM. Note that part of the contents of the world model is visible in the display in the upper left corner of Figure 2A. Figure 2C illustrates the interaction between the components of the system. Label (1) corresponds to SQL queries to ABM SQL, and (2) to replies that are managed by the Autobiographical Memory interface module (ABM C++). A link from the spoken language processing and supervisor to ABM C++ (3) allows the user to interact with ABM SQL related to action status, and to Memory content (4). The ABM reasoning function requests and receives content from the ABM (5–6), and extracts regularities from the encoded experience. ABM C++ requests and receives state data from OPC (7–8) to populate the ABM SQL. Final response of ABM Reasoning is provided to the supervisor via (9).

Again, the memory component of the ABM (ABM SQL) is made up of the episodic memory—a veridical record of experience, and the semantic memory which contains learned semantic properties, like the meaning of “north,” that are extracted by ABM reasoning. The structure of the episodic memory storage is illustrated in Figure 2D. When the robot stores an event in its episodic memory, it uses an instance of this structure. We consider an example where the iCub moves a toy from the center to the left of the table. In the main SQL table information related to the context is stored: an instance number (the id of the memory), the time, a name (tag, here “put”), an id of action (for internal request), the type of event (sentence, action, recognition, here: “action”), and whether the event begins or ends. The table contentArg stores more concrete information about the arguments of the event (agents involved, objects involved, arguments of the action, i.e.,: “predicate: put—agent: iCub—object: toy—recipient: left”). The table contentOPC will store the information relative to the state of the world at the moment of the memory, from the perceptual working memory of the system, the OPC (object properties collector). Specifically this includes the objects present with their cartesian position, orientation, color, and dimension, as well as perceived relations, e.g., “toy is at location left”). Such a snapshot is taken before and after each action. By comparing the state of the world before and after, the ABM reasoning extracts knowledge related to pre- and post-conditions of actions, and thus can learn action definitions from experience.

**Figure 3:**
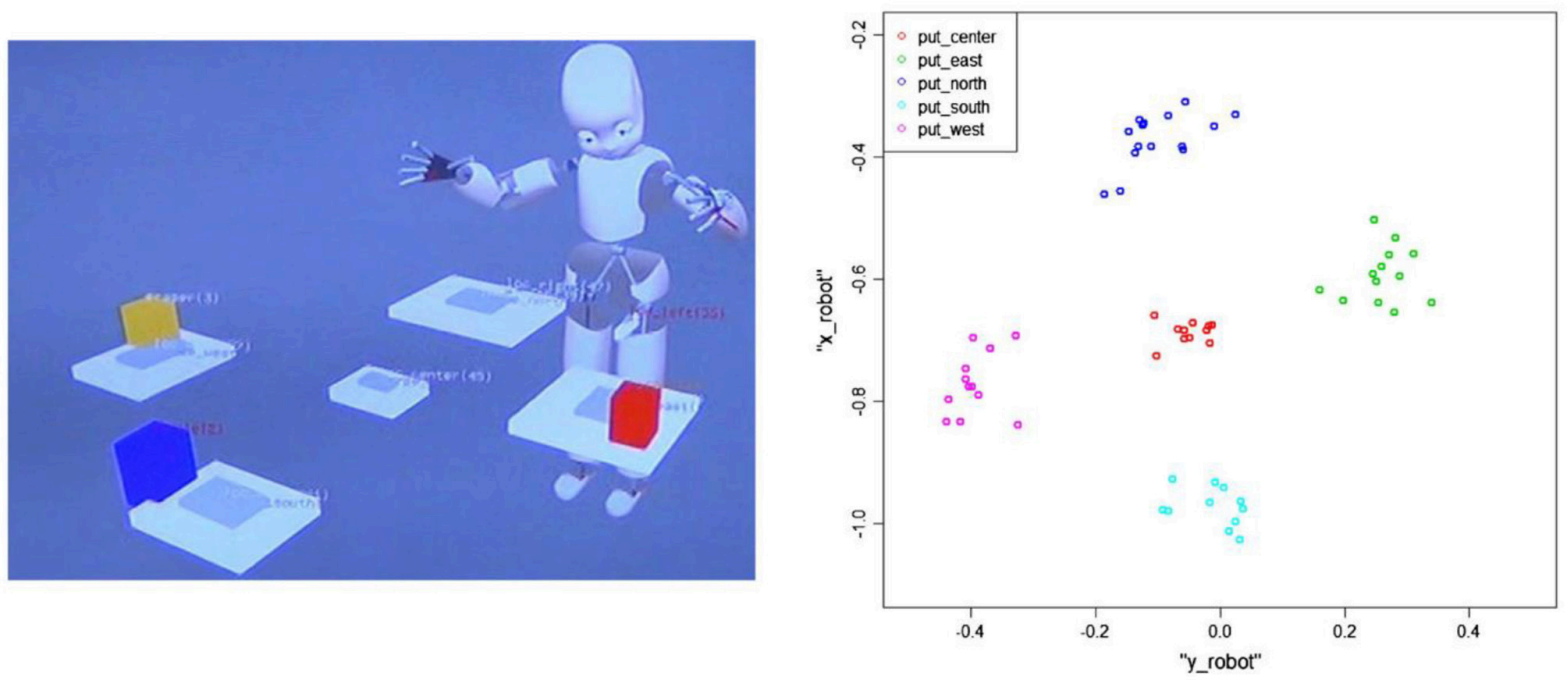
Results of learning spatial locations and actions. **(Left)** iCub's internal representation of learned locations in its working memory, the Object Property Collector (OPC). Gray boxes correspond to learned spatial locations, and colored boxes correspond to recognized objects on the table. **(Right)** Clustering of spatial locations associated with 5 named locations in the ABM that allow learning of the generalized location associated with each name.

An example of such knowledge is illustrated in Figure 3. In the left panel we see the iCub within its mental representation of itself and its physical environment (as represented in the OPC). Gray boxes are spatial locations that have been learned through association of clustered points, illustrated on the right, and corresponding spatial names that have co-occurred during interactions with the human. Such learning occurs, for example, when the human puts an object at one of these locations and says “I put the block North,” etc. Accumulation of this experience in the ABM allows the ABM reasoning process to collect correlation statistics on co-occurrence of words and spatial locations, to learn these spatial terms. Further reasoning allows the system to learn the meaning of the action “put” when it is associated with placing objects at these learned locations. The ABM thus provided a progression of learning from locations, to actions directed to learned locations, to shared plans (described below).

### Reasoning capabilities for knowledge generation

Given the architecture illustrated in Figure 2, the robot can store extensive records of its experience in interacting with the human. However, in order to adapt to novel situations a system must have memory of its experience, but this is not sufficient. The system must be able to extract regularities from specific cases, that can then be applied to the general case. We thus developed a set of reasoning capabilities that operate on the contents of the ABM (Pointeau et al., 2013, 2014; Petit et al., 2016). Here we explain the concept of the recursive algorithm developed for this processing.

**Figure 4:**
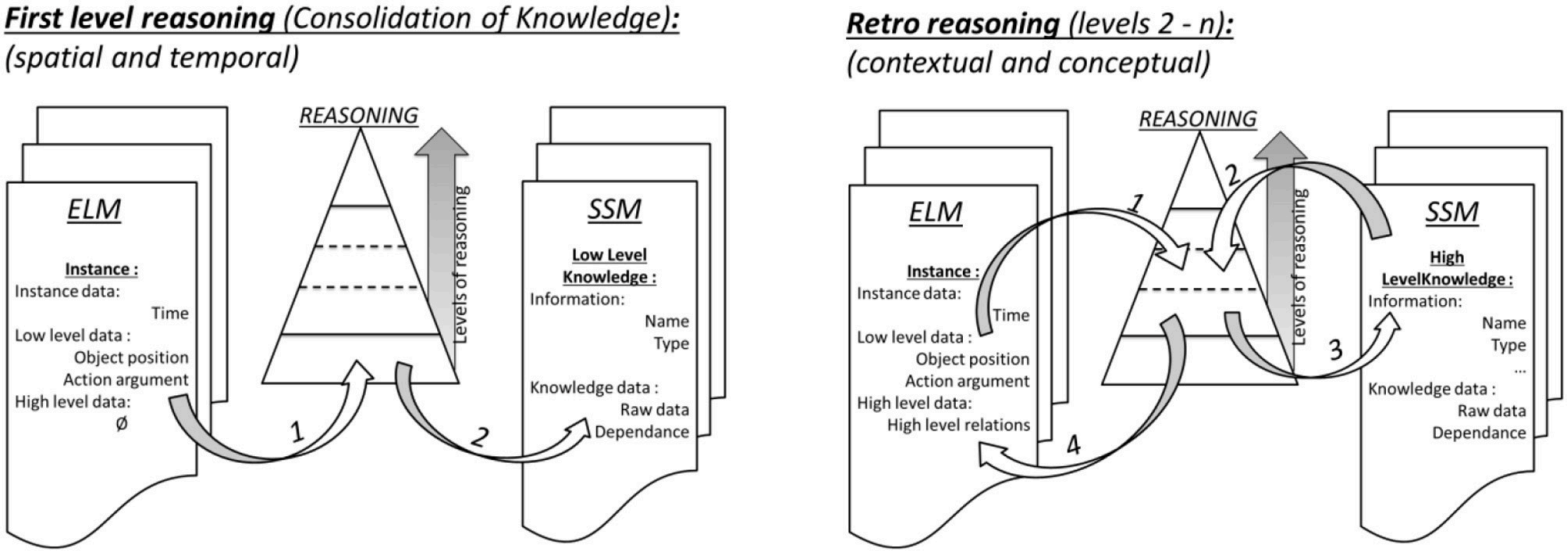
Episodic-Like Memory ELM, Synthetic Semantic Memory SSM. Recursive reasoning for creating knowledge. **(Left)** First level of reasoning: the iCub retrieves the events in the ELM, and discovers the associated meanings, by using clustering to detect regularities that co-occur with spatial and temporal terms. **(Right)** 2nd to nth level of reasoning: Retro-reasoning: Once the first level of knowledge has been created, it can be reinjected back into the EML, and the next levels are developed in a loop that will use the previous level of knowledge created as new data, to create the next level of knowledge. From Petit et al. (2016).

Consider how the meaning of the spatial term such as “east” is learned. The robot will first cluster episodes of its memory to create a first level of knowledge based on his experience. For example, each time the action “put object east” is performed, the final coordinate of the object is in *x* positive, *y* near zero. ABM reasoning collects statistics from all cases where “east” is mentioned, and finds this spatial regularity, and thus creates knowledge of “east” which indicates a location with these constraints. A second level of reasoning then uses this knowledge to create higher level of knowledge: retrospectively the robot adds this information to the ABM under the format of a new relation such as “object-is-east” (see Figure 4).

A third level of reasoning will once again retroactively use this enriched experience to create a new higher level of knowledge about actions: “*The consequence of the action* put-object-right *is that the* object *is at the location* right”. In addition to knowledge of actions, the clustering algorithms create knowledge about space (meaning of words such as: “North, South, East, Center, Left, Right…,)” time (meaning of words such as: “Before, After, Slowly, Quickly”) language (meaning of pronouns or agent's name: “I, You, John…”) drives (which action to use in order to satisfy a drive such as: “iCub wants the toy”).

These different levels of reasoning are summarized in Figure 4. This reasoning employs a consolidation capability, where contents of the episodic memory are re-played and the regularities (e.g., the spatial coordinates associated with the term *east*) are analyzed and encoded in the semantic memory. This replay requires representation in the system's working memory, which is referred to as the OPC. In order to keep the actual world view in the OPC separate from this mental simulation, we were required to implement a second “mental” OPC (MOPC). As will be seen below, this MOPC for replaying experience will have interesting new uses in the development of different levels of self.

Now, given the capability to interact with objects and people in the world, and this ABM capability, can a robot develop aspects of self? We now review a series of studies that attempt to answer this question.

## Toward neisser's four levels of self

While we tend to consider the self as a unitary object, Neisser suggested that in fact the self consists of essentially different selves, that differ in their origins and developmental histories (Neisser, 1988). His resulting analysis and decomposition of self into the ecological, interpersonal, conceptual, temporally extended and private selves had a major impact on developmental psychology, and in the way we understand the development of self. Indeed, his specification of these levels of self can be considered as requirements on the construction of a self for developmental robotics. Here we review research that attempted to take these requirements and determine if robotic systems could be developed that would meet these requirements.

In the developmental robotics perspective, capabilities of the system develop: they are structured through the interaction of the system with the environment and other agents. Clearly, however, one must start with some “core” capabilities, as does the child (Spelke and Kinzler, 2007). This forces one to address a fundamental question in cognitive development, and neurorobotics. That is, what is built into the system, what is the point of departure, and what is acquired or developed? From the perspective of the studies reviewed below, the answer to this question is specified by the architecture in Figure 2. That is, the robot has a perceptual system to observe actions and a motor system to perform actions (Lallée et al., 2011), a language system to understand and produce simple language related to action (Dominey and Boucher, 2005b; Hinaut and Dominey, 2013; Hinaut et al., 2014, 2015), and the ABM infrastructure described above. While these are minimal requirements, one can also consider that these could be embedded as part of a more complete system (Lallee and Verschure, 2015).

In the human, the neural infrastructure for autobiographical memory is significant (Svoboda et al., 2006), indicating its importance in human cognition. We also made a significant investment in the ABM system that we developed for the iCub, as described above. We now review a series of studies that together demonstrate how aspects of each of Neisser's four levels can be achieved with the support of ABM.

### Ecological self

The first level of self that we will approach is the first one to appear in human development as described by Neisser, the ecological self. “The ecological self is the individual situated in and acting upon the immediate physical environment. That situation and that activity are continuously specified by visual/acoustic/kinesthetic/vestibular information. […] infants perceive themselves to be ecological selves from a very early age.” (From Neisser, 1995, p. 18). Neisser places this level of self as the first self-developed by the child, and the more prominent in development. Indeed, this level refers to the relation between the person as a physical body, and his direct environment. Before going further in the understanding of the world, other agents, and trying to generate higher level knowledge, one has to develop a sense of self related to one's own physical body. Our first step in this direction was to allow the system to learn, from its own experience, a forward model of itself in action, and a body schema.

#### EcoSelf 1: simulating the consequences of actions from experience

In child development, sensitivity to perceptual primitives, including contact and motion, can be used to construct and detect higher level concepts like goal-directed action (Mandler, 1992). Based on this principal, we developed algorithms that extract the meaning of actions based on changes in state coded in terms of perceptual primitives like contact and motion (Dominey and Boucher, 2005a,b; Lallée et al., 2009, 2011, 2012; Pointeau et al., 2014).

**Figure 5:**
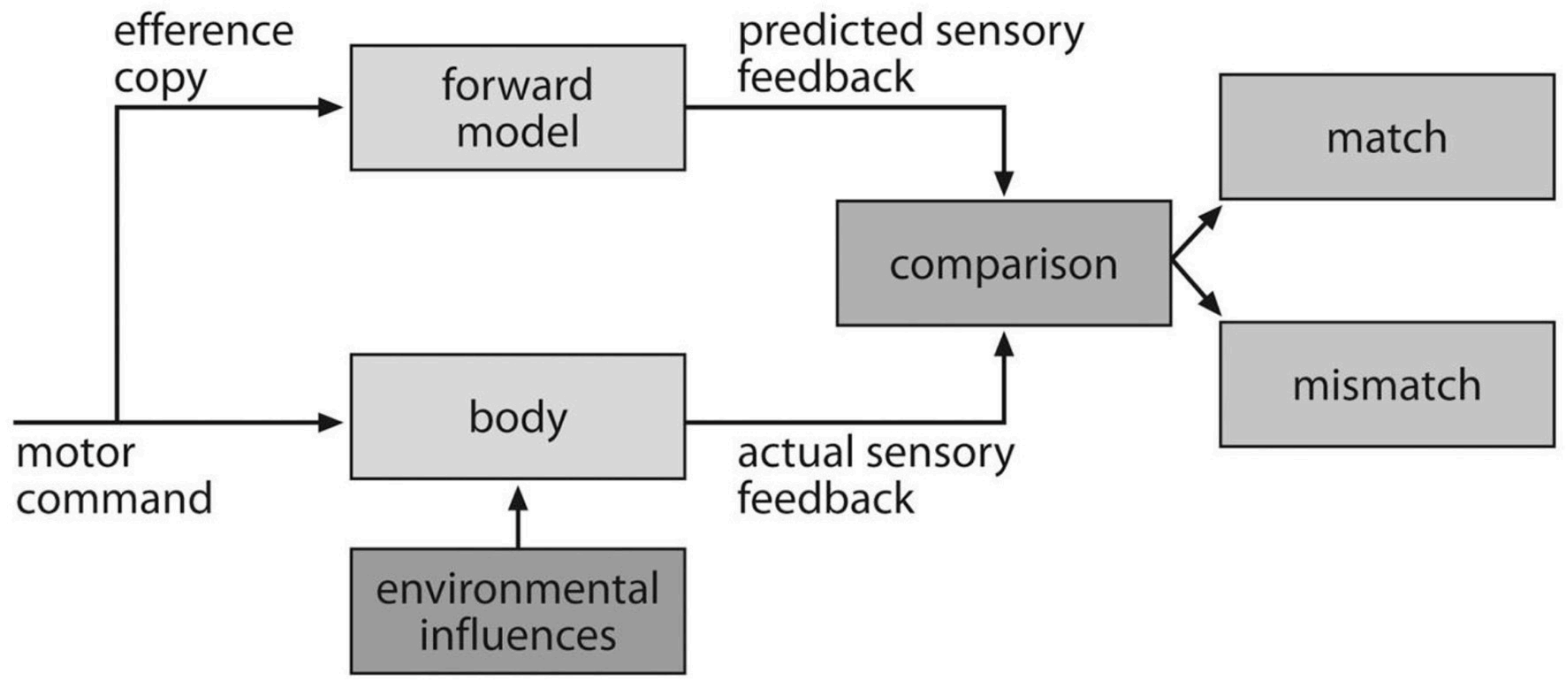
Illustration of the forward model in the context of the motor control. The motor command is sent to the motor command system and to the internal model. Subsequent comparison allows the system to determine if the grasp was correctly executed (from Bubic et al., 2010).

This self-experience provides the basis for knowledge that allows the system to predict the outcome of its own action, an important component of the ecological self. Indeed, the ability to create and use internal models of the body, the environment, and their interaction is crucial for survival. Referred to as a forward model, this simulation capability plays an important role in motor control (Wolpert et al., 1995). In this context, the motor command is sent to the forward model in parallel with its actual execution. The results of the actual and simulated execution are then compared, and the consequent error signal is used to correct the movement, illustrated in Figure 5. Interestingly, we demonstrated how experience of its own action coded in the ABM allowed the iCub to construct a forward model of pre-conditions and outcomes of these actions (Pointeau et al., 2013).

**Figure 6:**
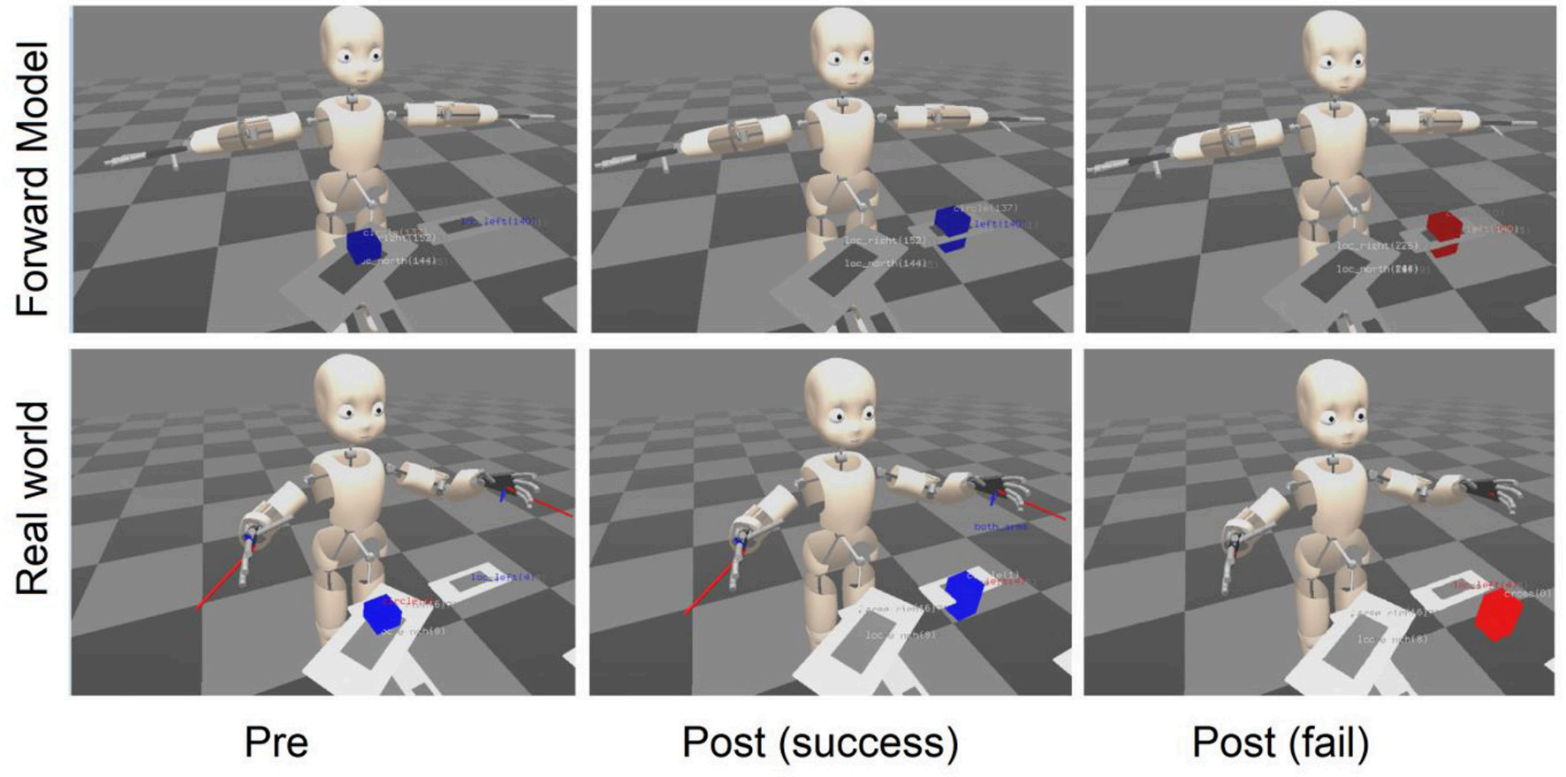
Use of the mental simulation as forward model in the case of the action “put-object-left” where center column in blue is a success and right columns in red is a failure. Mental image (Forward Model) and actual physical state (Real world) before and after the action in each case are represented. The action to perform is to put the object to the left of the robot in the delimited location. Pre—indicates the state prior to the action. Post (success) shows the predicted and observed view of the outcome that match. Post (fail) shows that the predicted forward model outcome does not match the actually perceived real world outcome, thus indicating that the action failed. (modified from Pointeau et al., 2013).

During interaction with objects, experience was coded in the episodic memory of the ABM. Then, using the ABM reasoning described in the section on Reasoning Capabilities for Knowledge Generation, regularities corresponding to the pre- and post-conditions of actions were extracted and coded in the semantic memory of the ABM, thus forming the forward model (Pointeau et al., 2013). This knowledge of the pre- and post-conditions provided the robot a capability to predict the consequences of its actions before actually performing them, a forward model, using a mental world to simulate actions (Figure 6). Recall that the real-time world state is contained in a form of working memory called the OPC, linked to the sensors and effectors of the robot. As described above, in order to consolidate memory we created a separate Mental Objects Properties Collector (MOPC) where actions can be replayed during consolidation. We thus exploited this MOPC to allow the system to simulate the consequences of actions, while not interfering with ongoing real-time perception. Indeed, as illustrated in Figure 6, the predicted outcome in the MOPC was compared with the actual outcome in the OPC (Pointeau et al., 2013), in order to allow the robot to determine if it had correctly performed an action. A more extensive treatment of this learning of forward models can be found in Dearden and Demiris (2005).

As illustrated in Figure 6, this learned forward model could thus be used in a control loop in order to compare action outcomes predicted by the model, with actual outcomes. Part of the novelty of this research was that the information used to allow the simulation of action was purely acquired from experience. In this sense we can say that the simulation capability is embodied in the sensorimotor experience of the iCub robot. In a related approach, Vernon et al. (2015) propose a joint episodic-procedural memory for goal directed internal simulation and prospection. This provides a viable method to approach the integration of procedural and episodic memory as a joint perceptual-motor system.

#### EcoSelf 2: body schema and mental imagery

With the help of a forward model mechanism, we thus provided the iCub with a way to interpret the consequences of its action upon the world. However, we could further improve the status of the ecological self by providing the iCub with a representation of its body schema. While the forward model described above is a form of embodied representation, it remains symbolic. How can more embodied representation be formed? Recent research in neuroscience can provide responses. Damasio and colleagues have proposed an embodied model of meaning representations based on convergence-divergence zones (CDZs) (Damasio, 1989; Meyer and Damasio, 2009). In this framework, sensory and motor representations are initially segregated in modality specific cortical areas. These areas send converging projections to integrative hubs that form CDZs. During learning, information from specific modalities converges in the CDZs. During recall, activation from one modality converges to the CDZ. Activation of this CDZ then, by *divergence*, will activate the other modality specific representations, thus recalling the original multi-modal experience (Damasio, 1989; Meyer and Damasio, 2009). We have recently demonstrated the possible role of such CDZ mechanisms in the anterior temporal pole and the temporal parietal junction of the human brain as people understand written sentences and visual images that depict human activities (Jouen et al., 2015). In the iCub system, because we have access to the different sensory and motor modalities of the robot, we should be able to create such embodied representations.

**Figure 7:**
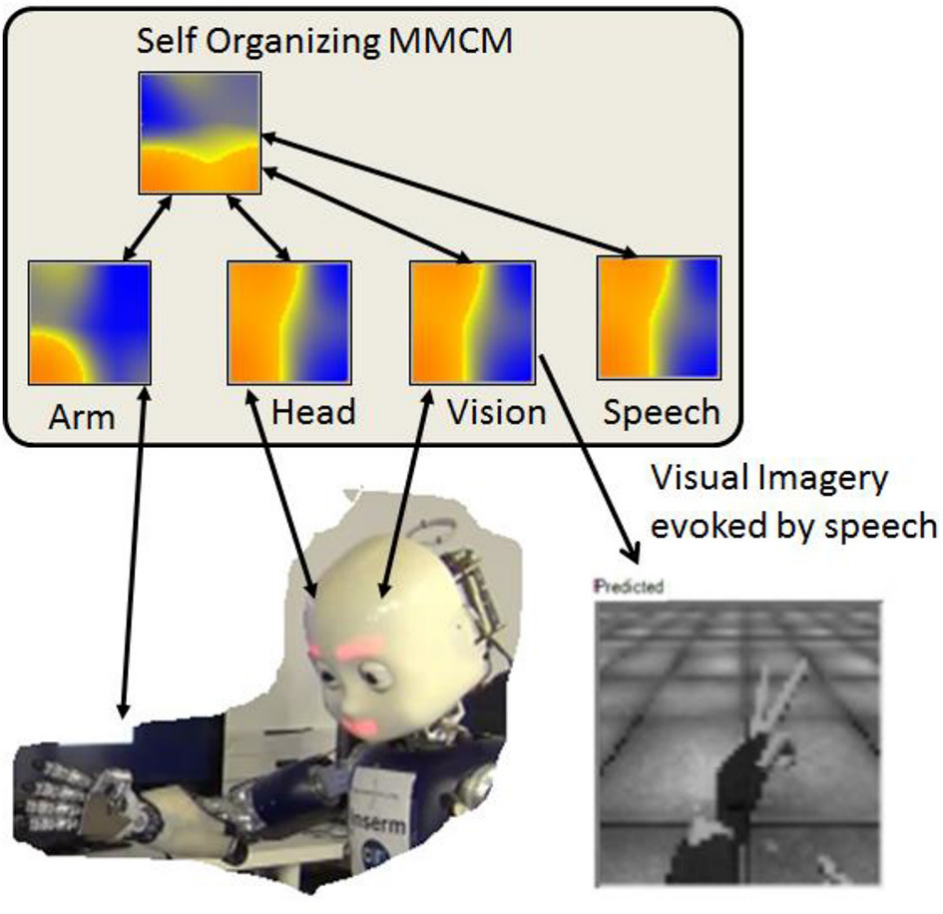
Body schema. Multi-modal convergence map model merging proprioception, vision, and language. Arm and Head are 50 × 50 maps taking as input the robot encoders (left\_arm = 16 degrees of freedom and head = 6); the speech is a 50 × 50 map fed by a string (converted to a vector of double). The vision is from the iCub left eye camera, gray scaled and cropped to a fovea area and rescaled to an experiment dependent resolution (from 15 × 15 to 320 × 240). These low level maps converge to the multi-modal convergence map. Then, activation of a single input map activates the MMCM which by divergence activates the other input maps. The image of the hand is actually reconstructed from input to the vision map from the stimulated MMCM. From Lallee and Dominey (2013).

To do this we modeled a set of sensory-motor cortical areas, and a CDZ area as self-organizing maps (SOMs) (Kohonen, 1998) that we call multi-modal convergence maps (MMCM). These maps were organized in a convergence-divergence architecture as illustrated in Figure 7. We postulated that the iCub should be able learn the association between different modalities of his body (vision, audition, and proprioception). To do so, the physical sensation of the robot (joint values of the head, eyes, arms, and hands) and the vision of the robot were associated using the MMCM based on the hierarchical CDZ Framework developed by Damasio (Damasio, 1989; Damasio and Damasio, 1994), and illustrated in Figure 7. A convergence map was able to learn the link between the different modalities by looking at its body (vision input) while at the same time feeling it (head and arm proprioceptive input). By training these maps during physical action, the system was able to learn a topological representation of the sensory-motor space that could be used to control the robot and to provide mental images of the robot's representation of its body. That is, after learning the maps that associate the word “scissors” with the scissors hand posture, the head posture to look at the hand, and the camera image of the hand, hearing the word “scissors” would, by convergence and divergence, reactivate the motor map to generate the posture, and reactivate the visual map to create a simulated mental image, as illustrated in Figure 7.

In the context of Neisser's ecological self, we thus consider the body schema as a neural representation that organizes multimodal representations (e.g., vision, proprioception, touch) into a coherent functional whole for behavior. SOMs were used to allow the iCub to learn its body schema (Lallee and Dominey, 2013). Figure 7 illustrates how through experience, the iCub has learned the association between a proprioceptive posture, the camera image of the hand in that posture, and the spoken name of that posture (in this case the word “scissors” in the “rock, scissors, paper” game). In this body-schema context, Hoffmann et al. (2010) note that in the majority of studies on the development of robot body schema's typically only vision and proprioception are used in the multimodal context. The use of vision, proprioception and simple language demonstrates the extensibility of such multimodal approaches.

These two systems: the forward model implemented in the ABM, and the MMCM body schema can provide to the robot a way to interpret the consequences of his own action upon the world and upon itself, thus constituting a first step toward development of the ecological self. These capabilities are completely conditioned upon the experience that the robot has acquired, and allow the robot to better understand his body in an ecological way (as described by Neisser). Schillaci et al. (2016a) review related studies of sensorimotor exploration in the creation of internal body representations, and sensorimotor simulation. They provide convincing arguments that exploration is a key ingredient in creating the context in which these representations can be formed. This is quite complimentary to our approach where the learning is more actively guided by the human partner. Schillaci et al. (2016b) likewise exploit forward model learning, here in the case of predicting ego-noise for noise cancelation.

After this demonstration that ABM can contribute to a forward model for the ecological self, the next step in Neisser's hierarchy of self is the ability to understand multiple agents and their respective roles: the interpersonal self.

### Interpersonal self

What are the capabilities that allow one to react in a direct social interaction? From a very young age, humans are able to detect if someone else's actions or movements are directed toward themselves or not (Gergely et al., 1995; Woodward, 1999). This contributes to constitute what Neisser qualifies as the Interpersonal Self. “The interpersonal self is the individual engaged in social interaction with another person. Such interactions are specified (and reciprocally controlled) by typically human signals of communication and emotional rapport: voice eye, contact, body contact, etc. This mode of self-knowledge too, is available from earliest infancy” (From Neisser, 1995, p. 18). Thus, the interpersonal self corresponds to the individual engaged in social interaction with another individual. This includes communication via gaze, and language, but in the repertoire of human behavior, cooperation can be considered a hallmark of interpersonal self.

Cooperation requires the construction of a shared plan which is a representation of the coordinated actions of the self and the other, and how they are undertaken to achieve the common goal of both parties (Tomasello et al., 2005). We can thus consider that the ability to cooperate with others demonstrates the successful functioning of the interpersonal self, as it requires a coordinated interaction with the other. In our approach to the interpersonal self, we first examined how mechanism for interpreting one's own actions could be applied to understanding others. We then extended this to determine how other aspects of interpersonal communication including language and gaze contribute to cooperative interaction.

#### InterSelf 1: understanding other's action

The first step into the Interpersonal Self was the simple extension of the forward model capability developed for the Ecological Self, but applied to another agent's action. Using the forward model, provided by the ABM, to simulate the action of others allowed the iCub to detect a mismatch between the declaration of the action of the agent, and the realized action. By using the forward model of the self (implemented as described above) in the MOPC, the system was able to simulate the expected outcome of another agents action, and then to compare this prediction with the actual outcome. This allowed the iCub to determine when an agent is telling the truth or not. Thus it was quite interesting that the mental simulation for self can be used directly to simulate another agent (Pointeau et al., 2013).

Related work by Copete et al. (2016) demonstrates how such models can be learned. During action, sensory and motor signals were learned in a predictive model. During observation, the sensory signals could drive the sensory and motor predictor, thus allowing prediction of other's actions. In an interesting turn on these approaches, Hafner and Kaplan (2008) considered how sensorimotor maps that include the actions of others can be constructed during coordinated action, and can form the basis for recognizing coordinated joint action. This inclusion of the other in the self body map recalls the notion of the inseparability of self and other in action described by Merleau-Ponty (1967), “In the perception of others, my body and the body of others are coupled, as if performing and acting in concert” (Rochat, 2010).

#### InterSelf 2: foundation for shared plans in the autobiographical memory

The results reviewed in the previous section allowed the robot to engage in a simple form of interpersonal interaction at the level of single actions. We sought to extend this to multiple actions in shared plans. Shared plans correspond to representations created and negotiated by two agents that allow them to act together in a coordinated way to achieve their shared goal (Tomasello et al., 2005). Because of the crucial role of shared plans in cooperative behavior, we focused on the implementation of shared plan learning and use in the context of cooperative human-robot interaction. Our previous research demonstrated that indeed, a robot equipped with the ability to learn and use shared plans could successfully learn new cooperative tasks and use the learned shared plan to perform the shared task with novel objects (Petit et al., 2013).

In this context of interpersonal self and cooperation, a crucial test for the ABM was to determine if it could be used to allow the creation, storage, and reuse of shared plans (Pointeau et al., 2014). We had already determined that it was sufficient for learning single actions and their arguments [e.g., put(iCub, eraser, North)]. A shared plan is sequence of actions, each of which has arguments. In this view, a shared plan is a more complex action that takes arguments, which are then passed on to the component actions.

Table 1 illustrates a readable form of a shared plan that was learned by the iCub and represented in the ABM. The learning of the shared plan was invoked by a command “You and I will play music with the drum, the guitar and the keyboard.” The system recognized that it did not know such a shared plan and asked to learn it. The human then instructed the iCub with a mixture of saying actions, e.g., “You put the drum north” and demonstrating actions by performing the next action in the shared plan, so that the iCub could recognize the action that was to be performed next. The system performed argument matching between the announced shared plan, and the subsequent component actions. In the two sentences above, “drum” matched in the shared plan command, and in the first action that was specified in the plan. This allows the system to generalize, so that the specific object drum, guitar and keyboard could be replaced by any objects in future invocations of the shared plan. This is a form of generalization to create variables in a shared plan (Dominey et al., 2007). This allowed the iCub to learn shared plans in a single trial, and then to generalize to performing the same shared plan with new objects and new agents^1^.

**Table 1:** Readable representation of a shared plan for a music game that was learned by the iCub via demonstration and spoken language.

Music (Agent1, Agent2, Object1, Object2, Object3) {
Default: Object1 = Drum,
Object2 = Guitar, Object3 = Keyboard,
Agent1 = Human, Agent2 = iCub
put(Agent1, Object1, North)
put(Agent2, Object1, South)
put(Agent1, Object2, North)
put(Agent2, Object2, West)
put(Agent1, Object3, North)
put(Agent2, Object3, East)
}

**Figure 8:**
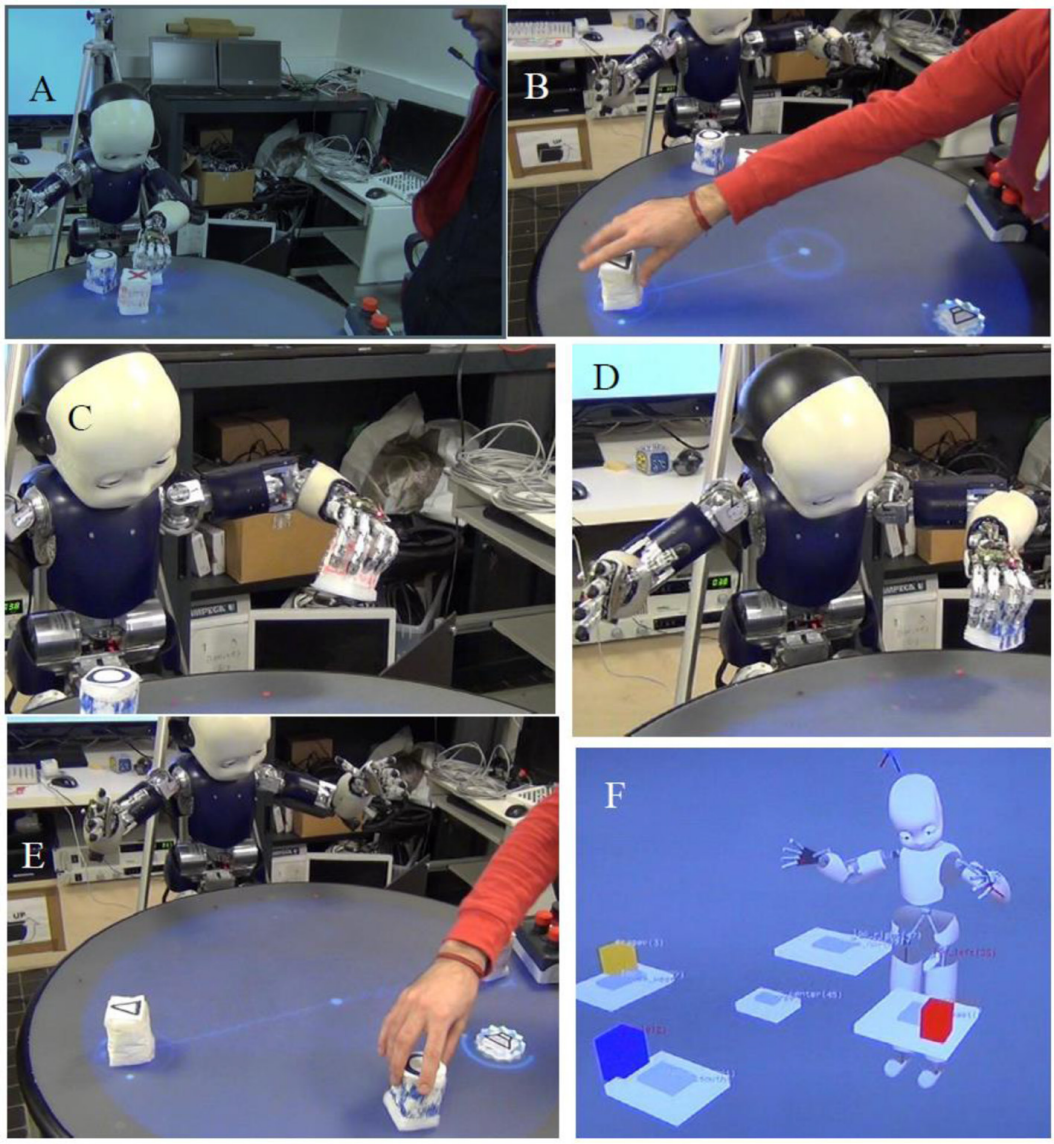
Different steps of the iCub during the execution of a shared plan for a musical came. **(A)** Initial configuration of 3 elements. The robot places the object 1 (white and yellow) to the location North. **(B)** Human takes the object and places it on the location West. **(C)** The robot places the object 2 (red) to the location North, for the human, who then puts it to the location East (not displayed). **(D)** The robot places the object 3 (blue) to the location North. **(E)** The Human takes the object 3 and puts it to the location South. **(F)** Final internal representation of the objects on the ReacTable (a musical table) to produce the song as the joint goal of the shared plan. From Pointeau et al. (2014).

Figure 8 displays the progress of a shared plan within the iCub and a Human agent. The shared goal is to produce a song using the ReacTable(TM) (a form of music device where different objects produce different sounds, depending on their location and orientation). Actions that have been learned in the ABM are structured using language into a turn-taking plan that allows the human and iCub to achieve a goal, in this case playing a song.

Shared planning likely mobilizes all of the available interpersonal self capabilities. To address this, in Lallée et al. (2013), we extended this work in cooperation, and evaluated the psychological plausibility and efficiency of a human-like dyadic interaction based on shared plans expressed through gesture, gaze and speech. We confirmed the prediction that optimal cooperation between a naive human and robot requires that the robot has a shared plan, and that it communicates this shared plan through all modalities available including spoken language and gaze.

The notion of interpersonal self is embedded in the social interaction with other, and the ability to understand the social clues provided by other. Crucially, autobiographical memory is required in order to accumulate and exploit relevant experience (Nelson, 2003b). Here we've reviewed how the ABM allows the robot's experience to be structured and re-used in the service of the interpersonal self, starting with the mental simulation of other's actions. Using the ABM related to its own actions, the iCub was able to create a forward model, and then use that model to predict the outcome of the human's actions. We then saw how the ability to represent these single actions could be extended to highly pertinent notion of shared plans. Again, the ability to cooperate toward a shared goal using a shared plan is one of the high points of human interpersonal behavior (Tomasello et al., 2005). By using the ABM to chain actions into sequences, the ability to represent shared plans was achieved. These shared plans represent a concrete interpersonal link between the robot and the human. This relationship between an individual, robot or human, and its social environment is a part of what Neisser defined as the interpersonal self. Again citing Neisser (1995) on the involvement of the interpersonal self: “That attribution is justified only if the infant looks for—and finds—the social consequences of its own social behavior” (p. 20). The shared plan is a concrete example of this.

### Conceptual self

In Neisser's words, “The conceptual self, or self-concept, is a person's mental representation of his/her own (more or less permanent) characteristics. That representation which varies from one culture to another as well as from one person to the next, is largely based on verbally acquired information. Hence, we can think of it as beginning in the second year of life,” Neisser (1995), (p. 18). The conceptual self corresponds to the notion of “having a concept of himself as a particular person.” We are distinct agents of the same environment. We have a set of “intrinsic properties” that define us, and that distinguish us from our neighbor. Neisser goes further by highlighting the contribution of experience in the development of conceptual self: “Where do cognitive models come from? Like all other theories, they are based on a mixture of instruction and observation. We acquire concepts from our parents and our peers and our culture, and in some cases from reading and schooling as well” (Neisser, 1988). In this framework defined by Neisser, the experience of instruction and observation must be stored and organized in order to contribute to the conceptual self. We will review how the robot can use the ABM to store and organize experience that can become conceptual knowledge of its own capabilities. We then consider conceptual distinctions between self and other.

#### ConceptSelf 1: reasoning based on experience

We have seen how the experience that the robot accumulates can be consolidated into rule-like knowledge about the pre- and post-conditions of action. Here we make a link between this embodied learning, and powerful reasoning capabilities from artificial intelligence. One of the long-term strengths of research in artificial intelligence has been the development of reasoning systems that can exploit expert knowledge in well-defined task domains. A major challenge in this approach is to acquire the expert knowledge in a format that can be exploited by the AI planning and reasoning system. Interestingly, the iCub, equipped with the ABM and the resulting capacity for action representation with pre- and post-conditions, is well-suited for generating expert knowledge directly from its experience. This can be useful both at the interface of AI and robotics, and in developmental studies. For example, as in human development, the acquisition of knowledge at one level requires the consolidation of knowledge from a lower level. How is accumulated experience structured so as to allow the individual to apply this structured knowledge to new situations? As illustrated above, we have begun to investigate how a robotic system that interacts with humans can acquire knowledge that can be formalized automatically, forming the expert knowledge that can be used for reasoning.

As reviewed above, in the development of the ecological self, through physical interaction with a human, the iCub robot acquired experience about spatial locations. Once consolidated, this knowledge was used in further acquisition of experience concerning the preconditions and consequences of actions. Now we can show how this knowledge was translated into rules that were used for reasoning and planning in novel problem solving situations (Petit et al., 2016). The ecological self can thus be extended to the conceptual self.

We performed experiments where users demonstrated moves in the context of a Tower of Hanoi task. Because the robot could not perceive stacked objects, we made a variation called the Table of Hanoi, with small, medium, and large objects to place, respecting the same placement rules, but putting objects next to rather than on top of each other. Using the action recognition described above, the system learned the constraints on where objects could be placed, for example, the medium object could be placed near the big object, on an empty spot, but not near the small object.

**Figure 9:**
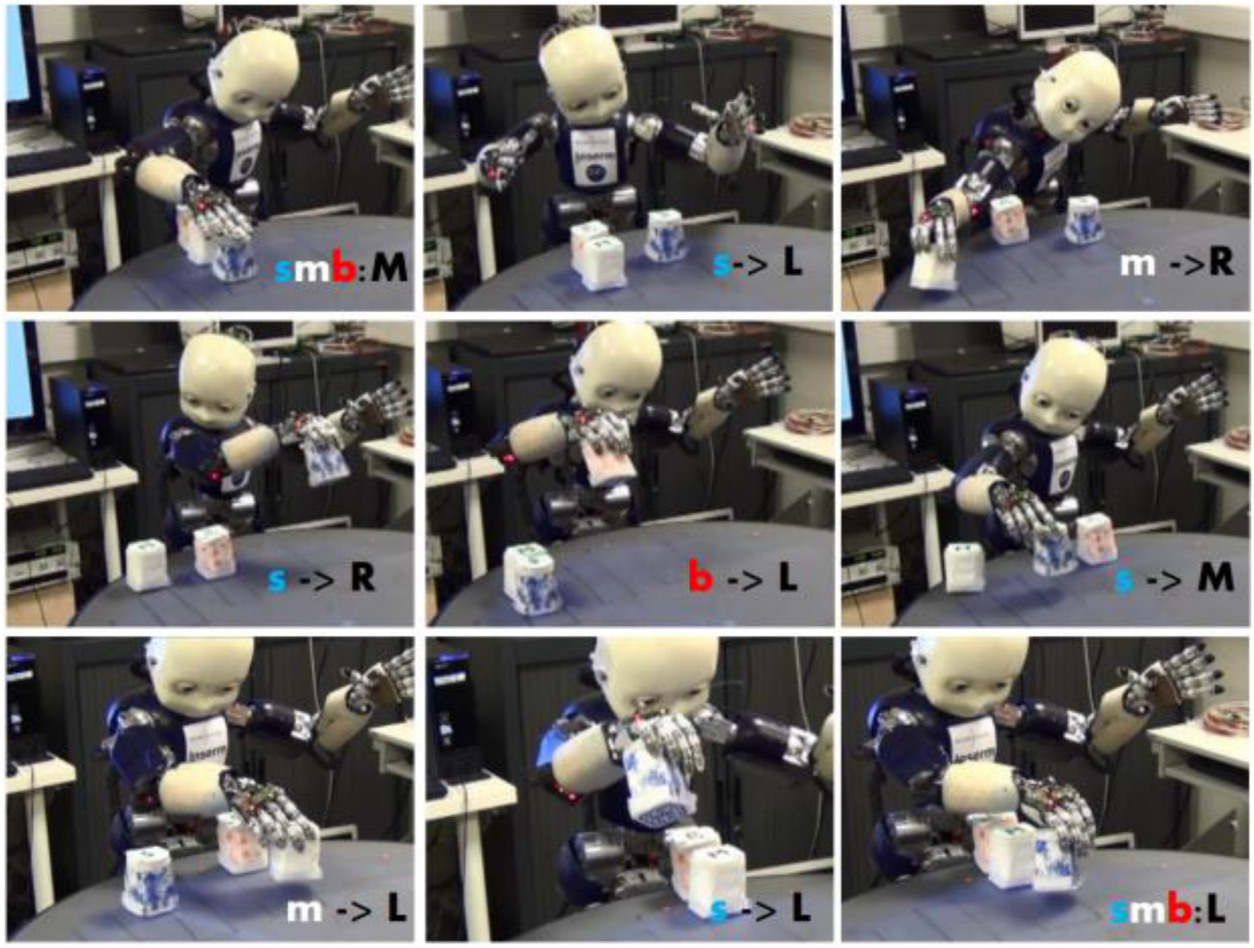
iCub solves a “Table of Hanoi” task, moving the small, medium and big object, from the Middle location to the Left location, after learning the rules that a larger object cannot go to a location where there is already a smaller object. These rules are used in a standard planner to allow the system to solve new instances of the task small-s, medium-m, big-b, middle-M, left-L, right-R. Intially all objects are at M, and the goal is to move them to L. From Petit et al. (2016).

After observing a human perform the different legal moves, the system automatically extracted the pre- and post-conditions for each move, and formatted them as illustrated in Table 2 in the PDDL format (McDermott et al., 1998). In the PDDL format, the contents generated directly from experience in the ABM was used with an off-the-shelf AI planner, to allow the iCub to then solve arbitrary configurations of the Table of Hanoi problem. An example problem solved is illustrated in Figure 9. This provided a first step for more flexible systems that can avoid the brittleness that has sometimes been associated with traditional AI solutions where knowledge has been pre-specified, instead using experience to acquire knowledge (Petit et al., 2016). The point here is that this self-knowledge becomes available for the system to organize goal directed behavior with forward looking plans in the context of the conceptual self.

**Table 2:** Rules of the “Table of Hanoi” problem, extracted automatically from the ABM based on observed experience.

**Rule**	**Preconditions**	**Post Conditions**
:action hanoi-small	(and (isAtLoc small ?from)	(and (not (isAtLoc small ?from))
parameters ?from ?to	(location ?to))	(isAtLoc small ?to))
:action hanoi-medium	(and(isAtLoc medium ?from)	(and (not (isAtLoc medium ?from))
parameters ?from ?to	(not (isAtLoc small ?from)	(isAtLoc medium ?to))
	(not (isAtLoc small ?to)	
	(location ?to))	
:action hanoi-big	(and(isAtLoc big ?from)	(and (not (isAtLoc big ?from))
parameters ?from ?to	(not (isAtLoc small ?from))	(isAtLoc big ?to))
	(not (isAtLoc small ?to))	
	(not (isAtLoc medium ?from))	
	(not (isAtLoc medium ?to))	
	(location ?to))	

*These extracted rules allow the iCub to successfully solve the ToH problem*.

#### ConceptSelf 2: mental representation of self and other

In the development of conceptual self we then considered the ability of the robot to use its self-representation to take someone else's point of view (developing one basic element of a theory of mind). We considered the famous Sally–Anne task (Baron-Cohen et al., 1985) to be a protocol that would allow us to evaluate these capabilities. The Sally–Anne experiment is designed to test the ability of a child to mentalize the mental state of other agents (and to create thus false beliefs). To do so, the child is placed in front of two dolls: Sally and Anne, a box and a basket that are both closed. Sally has a toy and puts it in the basket then leaves. Anne takes the toy from the basket and puts it in the box, and then closes the box. When Sally comes back, the child is asked: “Where will Sally look for the toy.” The expected answer is “in the basket.”

The human cognitive system allows us to travel in time and space where we can imagine possible futures, and relive and analyze the past. To do so, the system requires the ability to simulate itself and its activity in the world. We hypothesize that this simulation capability derives from the long evolved capability for forward modeling (described above in the section on ecological self) that was crucial for the ability of advanced primates to navigate through a complex world where real-time sensorimotor was crucial to survival. The ABM provides this capability, where through the accumulation of its own experience, the iCub can extract the regularities that define the pre- and post-conditions of its physical actions, and those of the human. This knowledge is then used to drive the mental simulation of action, which can actually operate faster than real-time, and generate predictions of expected outcome before the real movement is achieved.

This simulation can be used as a traditional forward model in the control sense, as we described above, but it can also be used in more extended time as a mental simulation or mental image that can contribute to higher cognitive function such as planning future actions, or even imagining the mental state of another agent. In this context, we exposed the iCub to a version of the Sally–Anne task, where the mental OPC was used to represent Sally's perception. We simulated the task as follows: the iCub and a human agent (labeled as Sally) were interacting across the ReacTable. The agent put an object on the location “left.” The iCub synchronized the mental OPC (MOPC) with the OPC. The agent left, iCub detected this and blocked the MOPC to the state of the world as it appeared while the agent was still present. Another agent arrived and moved the object to the location “north.” This corresponds to Anne moving the toy from the basket to the box.

**Figure 10:**
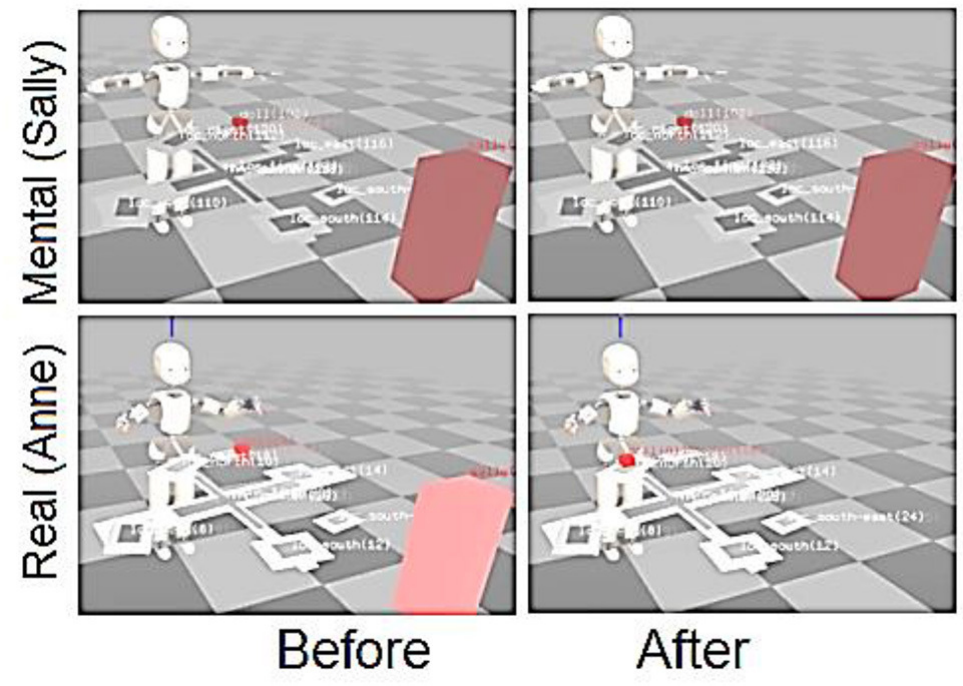
Contents of mentalOPC and realOPC in the Sally–Anne task. OPC stands for Objects Properties Collector and is a form of working memory. It stores everything the robot knows at the current time. In the “Before” column is represented the contents of both OPCs when the toy (the small red cube) has been placed at the first location (to the robot's left). The mentalOPC is the system's representation of what it and Sally have seen. In the “After” column, the Actual situation represents the contents of the realOPC after the toy has been moved (in front of the robot). In that same column the mentalOPC represents what Sally observed before she left. If this is maintained in memory, then it will persist after the world has been changed, and it can be used to mentalize about where Sally would look for the toy. From Pointeau et al. (2013).

When we interrogated the iCub concerning the differences between the real (Anne's perspective) and mental (Sally's perspective) OPCs at the end of the experiment, we obtained the following results as illustrated in Table 3. The iCub correctly believed that the Toy was at the location “column” and “north,” and that Sally was no longer present. The iCub represented Sally's belief, in the MOPC that was not updated, that the toy was still “left” (see Figure 10).

**Table 3:** Results from comparing the “true beliefs” attributed to the iCub in the real OPC and the “false belief” attributed to Sally in the MOPC.

3 entities changed
Entity: doll
robot_position_x −0.03
robot_position_y −0.28
robot_orientation_z −0.04
rt_position_x −0.12
rt_position_y −0.25
Entity: iCub
Beliefs added: doll is column doll is north (after)
Beliefs removed: doll is left Sally is isPresent (before)
Entity: Sally
The feliefs of sally didn't change, because she wasn't there.
Sally's beliefs are: doll is left

*The comparison indicates a significant difference, corresponding to the difference between the “false belief” attributed to Sally and the “true beliefs” attributed to the iCub*.

In the context of mentalizing and the false belief task, this research has significant potential impact. There is an ongoing debate concerning the nature of the mental processes that are required to take the mental perspective of another agent (Fletcher et al., 1995; Gallese and Goldman, 1998; Völlm et al., 2006; Corbetta et al., 2008). Our research provides insight into this question, by illustrating how a simulation capability that is directly derived from experience can be used to provide an agent with the basic representational capabilities to perform the false belief task.

However, it can be considered that the mere notion of “autobiographical memory” presupposes that the system must have a first person perspective (1PP), from which that memory is situated. The notion of 1PP is in fact a quite complex (Velmans, 1991; Baker, 1998; Vogeley et al., 2004). From the perspective of the current research, we can say that the robot has taken steps toward achieving a minimal form of 1PP in that it has developed an integrated representation of itself within the peripersonal space. This also comes back to the notion of ecological self as defined by Neisser, which is the individual situated in and acting on the immediate environment. What is currently missing with respect to these notions of self is a reflective capability, where the system reasons on a self-model as an integrated model of the very representational system, which is currently activating it within itself, as a whole.

This research makes a significant contribution to the cognitive systems research. It allows the iCub system to autonomously generate an internal simulation capability based on its own personal experience. This simulation capability can operate at the level of physical control, and at high levels of cognition including mentalizing about the belief states of others.

These two experiments (the high level reasoning and the Sally–Anne task) are both a significant step toward a conceptual self. But this reasoning about agents and the implication of actions can be seen at a larger time scale, with the temporally extended self.

### Temporally extended self

“The temporally extended self is the individual's own life-story, as one knows, remembers, tells, and projects it into the future. This requires aspects of the conceptual self, narratively organized episodic memory, and some understanding of continuity of persons over time. This notion of continuity applies at the individual level as the notion of psychological continuity” (From Neisser, 1995, p. 19). This is closely related to the notion of the narrative self (Gergen and Gergen, 1988; Bruner, 2009). A central aspect of this narrative self construction concerns how narrative provides structure to characterize the interaction between self and others over time. It is apparently this relational aspect that is crucial in defining the self (Gergen and Gergen, 1988; Nelson, 2003a). We can consider that the co-operative shared plans that the robot learns, through its interaction with the human, corresponds in some way to a first step toward this temporally extended self. Interestingly, this thus represents the “highest” level in our autobiographical memory hierarchy.

#### TempExtSelf 1: over four years accumulated experience

In November 2012 the Autobiographical memory system became sufficiently technically reliable that it was enabled and since then has been continuously encoding the experience of iCubLyon01. That encoded experience provides a record of the different kinds of interactions the robot has had, with different people, over time. Table 4 summarizes instances of agents, objects, and actions that were encountered, along with the number of sentences that accompanied them. At different periods, different types of experience were acquired. Learning about objects and the results of actions provided the basis for further learning about how to combine these actions into shared plans—shared plans that were learned and then re-played with people. This record can then contribute as part of the basis for the development of the narrative self (Pointeau et al., 2014).

**Table 4:** Summary statistics on data contained in the episodic memory of the iCub.

Instances:	18,838
Agents met:	21
Objects seen:	34
Sentences heard and said:	465
Actions known:	34
First instance:	2012-11-13
Last instance:	2016-05-23

*An instance corresponds to an entry in the autobiographical memory and can be triggered by any software component of the iCub (sensor, effector, reasoning)*.

#### TempExtSelf 2: shared plans and narrative self

The manipulation of shared plans is by itself a form of narrative self. Shared plans correspond to schemes that encode turn-taking interactions between the robot and a human, that allow them together to achieve a common goal. In the context of the narrative self, and temporally extended self, the shared plan is extended in time, but in addition, the robot can remember when it first learned how to engage in this shared activity, with whom, and then each subsequent instantiation of this activity. Via the use of an interface between the ABM and the language system, the robot can access its own history while interacting with a human. This provides a new dimension of shared experience over extended time, in the context of the development of the narrative self. We used the ABM system integrated into the Nao robot, in a series of experiments with naïve subjects teaching the robot how to repair a broken electronic card as a cooperative activity and shared plan with the subject. Again, this produced a significant data base in the ABM which could then be used as the contents in dialog between the human and the iCub, illustrating the narrative self. Here is a dialog excerpt from such an interaction:

H: When was the first time you learned repair electronic card?^*^
R: It was the 13 of March of 2014. You want to know more about it?
H: No. When was the last time you learned repair electronic card?
R: It was the 14 of June of 2014.
H: Who was there?
R: Marwin was there. You want to know more about it?
H: No. When was the first time you did hold?
R: It was the 13 of March of 2014. You want to know more about it?
H: No. When was the last time you did hold?
R: It was the 29, of April, of 2014. You want to know more about it?
H: How many times did this happen?
R: It happened 282 times. You want to know more about it?
H: Who was present?
R: Colas was present. You want to know more about it?
H: What have you done?
R: He taught me hold, and it worked. He taught me release, and it worked. He taught me Repair electronic card, and it worked. We did release, and it worked.
^*^H: human, R: Robot. From Sorce et al. (2015).

Creating the interface between the language system and the ABM allowed the first steps toward the narrative self. The robot could express its long term experience in language, extending the cooperative interaction capabilities. The past thus becomes available for interrogation by the human partner, in order to understand why a shared plan was learned in a certain way, who taught the shared plan, etc. (Sorce et al., 2015). The crucial next step was to allow narrative to enrich the experience of the iCub beyond what the iCub can directly perceive (Bruner, 1990).

Narrative enrichment takes place when narrative creates links between events that were not visible. When the human says to the iCub “I gave you the toy because you wanted it,” the human is introducing a causal link between the mental state of wanting, and the physical action of giving. This goes beyond the representation of the wanting and giving events that are coded in the ABM. In this context, we thus implemented a situation model (Zwaan and Radvansky, 1998) that encodes events and the relations between them and their component agents and actions in structured social interactions (Dominey et al., 2017). In parallel with these structured social interactions is the narrative structure that describes them. This led us to introduce the extension from grammatical construction to narrative construction. The grammatical construction is a mapping from sentence form to event meaning. The narrative construction is the mapping from a structured narrative, to the structured meaning representation in the situation model. While it is beyond the scope of this review, we note that the narrative construction, with its basis in experience coded in the ABM, can have powerful impact in the development of the self. In summary, narrative or stories, are vehicles for imparting social norms, folk psychological theories, to listeners (Hutto, 2009). Whereas sentences can describe isolated events, narratives can describe how events and agents fit together, including how things should and should not be done. Future research will investigate this link between experiences coded in the ABM, and narrative that gives that experience meaning. One thing is clear, the history of interaction encoded in the human ABM is crucial for the development of the narrative self (Nelson, 2003b), and in the iCub it also provides the basis for the temporally extended and narrative self (Dominey et al., 2017).

## Discussion

As suggested by Tomasello (1995); Tomasello et al. (2005), and others (Gergen and Gergen, 1988; Fivush, 1994; Nelson, 2003b; Rochat, 2009), a crucial aspect of human social cognition involves entering into self-other relations. This underlines the importance of the development of the self, then, as a pre-condition for entering into social relations. Taking the self as an object of study in developmental psychology, Ulric Neisser developed a systematic approach and identified five types of self-knowledge that we reviewed above. This elaboration of different aspects of self has been very productive in the developmental psychology domain, and it also provided a framework that would allow us to ask the question: can a robot develop a sense of self?

In a series of studies in the context of developmental robotics, we set out to implement the mechanisms underlying Neisser's different levels of self. Two important insights have emerged from this research. The first is related to the importance of memory, and particularly ABM, in the development of self. That is, the developmental robotics studies reviewed provide demonstrations of how ABM contributes to the different levels of self. This is potentially interesting from two perspectives. From the perspective of developmental psychology, the research reviewed here provides demonstration evidence in favor of the position of Bruner, Nelson Fivush, and others on the importance of autobiographical memory. From the perspective of developmental robotics, the research reviewed is of interest because it demonstrates a fruitful method for achieving robot cognition. The second insight is related to the interaction between the development of self, and social interaction. Interestingly, we see that there is a powerful synergy between the development of self and social interaction. That is, while social interaction requires a self, the development of self requires social interaction. Again, this observation is of interest both from the developmental psychology perspective and the developmental robotics perspective.

We have seen in this review how the ABM contributes to the emergence of different levels of self as described by Neisser: the Ecological Self, the Interpersonal Self, the Conceptual Self, the Temporally Extended Self. Extensive study of human cognitive development has concluded that autobiographical memory is a major component in the development of the self (Nelson and Fivush, 2004). Here we have reviewed how an ABM process can contribute to the different aspects of self as identified by Neisser, in the context of an adaptive cognitive system that allows the iCub humanoid to interact with people, and begin to engage in a self-other interaction. By encoding the preconditions and post conditions for actions performed by the agent, the ABM provided the framework for aspects of the ecological self. Consolidation of this experience allowed the extraction of representations of self action that provided the system with aspects of the ecological self, in terms of the effects of its actions in the immediate environment. Moving to the interpersonal self—the individual engaged in social interaction with other agents—this ability to model one's own actions could then be applied to others. The system could simulate the announced actions of another agent, and then compare this simulation with the actual result, thus extending the forward model to other agents. In a more advanced form of interpersonal self, the ABM was used to provide a capability for learning and using shared plans for cooperation in goal directed shared action with others (Pointeau et al., 2014). This constitutes one of the high points of primate social cognition (Tomasello et al., 2005), in the context of the interpersonal self.

The conceptual self—the acquired representation of one's characteristics—was also shown to be supported by the ABM. Indeed, the representation of self characteristics were used to allow the iCub to reason about what it was capable of doing, here in the context of a challenging cognitive task in the form of the Tower of Hanoi. This game can be characterized in terms of conceptual knowledge of rules about how the pieces can be moved. By observing legal moves being performed, the ABM encoded the corresponding preconditions and post conditions and generalized this to encode the rules of the game. We thus demonstrated that by using the ABM to allow the iCub to encode the conditions under which the different pieces could be moved, the resulting action rules could be used with an AI planner to allow the iCub to solve the Table of Hanoi task.

Finally, the ABM perhaps finds its most advanced aspect of self in the temporally extended or narrative self, precisely because the ABM is a temporally extended memory that is continuously being expanded in its contents. In 2016 over 18,000 distinct instances (experiences of actions observed or performed, state change, etc.) had been recorded since November, 2012. This memory can be accessed by language, allowing the system to say when it first learned something, with whom, how many times since then etc. (Sorce et al., 2015). This narrative memory is of particular interest, and is a focus of our current and future research.

It is worth characterizing what are the prerequisites for this processing. We previously suggested that shared planning could be developed based on 5 prerequisites: (1) object and agent perception, (2) perception of state changes (allows action perception), (3) ability to distinguish between self and other, (4) emotion/outcome perception, and (5) statistical sequence learning (Dominey, 2005). These mechanisms, plus a specific AMB and methods for operating on the contents of the ABM allow for the capabilities reviewed in this report. As mentioned, we find the need for one additional capability, which is an interface between the language system and the ABM, in the form of a situation model. This is required in order to explicitly represent narrative relations between events that are not accounted for in the ABM (Dominey et al., 2017). We believe that future research should address how narrative structure provides a framework for the developing infant to understand herself and others (Gallagher and Hutto, 2008; Gallagher, 2013), and for constructing their model of reality (Bruner, 1991; Fivush, 1994; Nelson, 2003a).

The ABM is not alone in the development of these different aspects of self: we also showed how a form of self-organizing maps can be used to provide the ecological self with a representation of the body schema based on Damasio's convergence divergence zones (Lallee and Dominey, 2013). Likewise, within our larger iCub framework, recurrent “reservoir” networks based on cortico-striatal neuroanatomy provided a learning capability for comprehension of grammatical constructions in the service of cooperative interaction (Hinaut and Dominey, 2013; Hinaut et al., 2014, 2015). The use of a mental work space (that we refer to as the OPC) allows the contents of the ABM in the form of action rules to be used to predict the outcome of one's own and other's actions and beliefs.

The current report reviews our efforts to map our research into the framework of Neisser, along with related work from the field. Table 5 illustrates how these studies map on to the four levels of self of Neisser. We focus on these dimensions of self, and thus acknowledge that related studies, for example in language grounding (e.g., Lemaignan et al., 2012) are not covered here. An extended review on related topics is also provided by Asada et al. (2009). Importantly, Lallee et al. (2015) highlight the importance of the interaction cues that allow a robot to be perceived as the other in these self-other relations.

**Table 5:** Summary of cognitive functions developed in the iCub and other robot systems, mapped onto Neisser's four dimensions of self.

**Ecological self**	**Interpersonal self**	**Conceptual self**	**Temporally extended self**
*Simulating consequences* (Dearden and Demiris, 2005; Pointeau et al., 2013; Schillaci et al., 2016b)	*Understanding others actions* (Hafner and Kaplan, 2008; Pointeau et al., 2013; Copete et al., 2016)	*Reasoning based on experience* (Petit et al., 2016)	*Four years of ABM* (Sorce et al., 2015)
*Body schema and mental imagery* (Lallee and Dominey, 2013; Schillaci et al., 2016a)	*Shared plans and Cooperation* (Dominey and Warneken, 2011; Lallée et al., 2012; Pointeau et al., 2014)	*Mental representation of self and other* (Pointeau et al., 2013)	*Learning shared plans* (Pointeau et al., 2014)
	*Embodied Grammatical constructions* (Hinaut et al., 2014; Mealier et al., 2016)		*Narrative self* (Sorce et al., 2015; Dominey et al., 2017)

The mapping of robot function into Neisser's levels does not imply that these levels have been definitively achieved, but rather that progress is being made in this context. In summary we have presented a set of studies that, taken together, help to illustrate how autobiographical memory can contribute to the development of aspects of self in a robot cognitive system. It is encouraging that the clear role of autobiographical memory in the development of self in child development (Nelson, 2003b; Nelson and Fivush, 2004) is reflected here in the development of self in a robotic cognitive system.

## Author contributions

GP and PD both made substantial contributions to the conception or design of the work; the acquisition, analysis, or interpretation of data for the work; drafting the work or revising it critically for important intellectual content; final approval of the version to be published; and agreement to be accountable for all aspects of the work in ensuring that questions related to the accuracy or integrity of any part of the work are appropriately investigated and resolved.

### Conflict of interest statement

The authors declare that the research was conducted in the absence of any commercial or financial relationships that could be construed as a potential conflict of interest. The reviewer ST and handling Editor declared their shared affiliation, and the handling Editor states that the process nevertheless met the standards of a fair and objective review.

## Footnotes

^1^Video demonstration of learning and executing a shared plan via ABM can be seen on Youtube at: http://youtu.be/GhlKHPZZn30 and http://youtu.be/eFeD-2S-V7M, respectively.
